# Effect of Frequency of Platelet Apheresis on Coagulation Function in Donors: A Prospective Cohort Study

**DOI:** 10.1007/s12288-019-01130-9

**Published:** 2019-05-06

**Authors:** Qing Feng, Faming Zhu, Chunyan Li, Beijie Guo, Jun Ye, Jiangtian Chen

**Affiliations:** 1 Blood Center of Zhejiang Province, 345 Wulin Road, Hangzhou, Zhejiang 310006 China; 2 Key Laboratory of Blood Safety Research, Zhejiang Province, Hangzhou, Zhejiang 310052 China

**Keywords:** Thromboelastography, Coagulation function, Regular donor, Platelet apheresis

## Abstract

The maximum number of plateletpheresis donation was permitted up to 24 times every year for each donor in China. This study was investigated the effect of donation frequency on coagulation function of the plateletpheresis donors. A total of 96 plateletpheresis regular donors (splitted into A, B, C groups with 32 donors each group. A was 6–11 times donations, B with 12–17 times and C with 18–24 times) and 32 first time plateletpheresis donors (D group) were analyzed. The coagulation reaction time (R), blood clot formation time (K), α-Angle, normal maximum amplitude (MA) were tested using thromboelastography instrument. Platelet (PLT) count was measured using a hematology analyzer. The ratio of the male to female were 23:9, 24:8, 27:5 and 24:8, the mean age were 40.7 ± 7.6, 39.8 ± 8.3, 40.2 ± 7.9 and 37.0 ± 9.3, and the platelet collection amount were 1.55 ± 0.37 U, 1.58 ± 0.38 U, 1.61 ± 0.33 U and 1.46 ± 0.31 U in the A, B, C, D groups respectively. There were significant difference in the values of the R, K, α-Angle, MA and PLT count between before and after plateletpheresis donation in each group (*p* < 0.05). However, the values of R, K, α-Angle, MA and PLT count in the before donation were not difference among the A, B, C, D groups (*p* > 0.05). Similar results were found in the after plateletpheresis donation. The number of the PLT was significantly correlated with the values of the R, K, α-Angle and MA (*p* < 0.05). However, the frequency of plateletpheresis were not significantly correlated with R, k, α and MA parameters (*p* > 0.05) using Spearman correlation analysis. The regular donation of apheresis platelets and the frequency of annual blood donation had no adverse effect upon coagulation function of the donors in China.

## Introduction

Platelet apheresis, or plateletpheresis is a process by which platelets can be collected but the other blood cells returned to the donor [1]. The platelet product from single donor plateletpheresis has high platelet concentration and less contamination with white and red blood cells, which could be greatly reduced the incidence of adverse reactions to transfusion [1]. It was known that the clinical efficacy of plateletpheresis is significantly better than that by manual collection, therefore it has been widely used in the clinical practice for many years [2, 3]. As the clinical use of plateletpheresis has been increased [3, 4], the contradiction between clinical supply and demand of plateletpheresis has become increasingly prominent due to a limitation of donors. To help address this in the USA, the FDA increased the number of maximum donations in a year from 12 to 24 times in 1988 [4]. However, this might be detrimental to donors [4, 5]. Then concerned about the effect of multiple platelet product donation on donor health, the FDA proposed restrictions limiting the number of platelet products donated in 12 months in 2005 [5].

The maximum annual donations was adjusted in 2011 in China, including the interval of plateletpheresis from 28 to 14 days, and the frequency of annual blood donation from 12 to 24 times *according to* “Health examination criteria of blood donor”. After this adjustment, the frequency of annual plateletpheresis in the donors has increased significantly in China. In 2016, the average frequency of plateletpheresis in our blood centre reached 3.5 times/year, of which 16% donors had more than 6 times apheresis platelet collections annually, and some even reached the maximum limit of 24 times. Katz et al. [6] reported that frequent plateletpheresis does not clinically significantly decrease platelet counts in donors in the USA. However, the data for the donation frequency on coagulation function of the plateletpheresis donors was limited. Now thrombelastography (TEG) is a widely technique used for coagulation function evaluation [7–9]. Here, in order to assess the impact of regular platelet apheresis on the blood coagulation and platelet function, we used TEG to analyze coagulation factor and platelet function in the apheresis platelet donors in China.

## Materials and Methods

### Subjects

A total of 96 regular plateletpheresis donors at the Blood Center of Zhejiang Province from July 2016 to July 2017 were included in this prospective cohort study. According to the frequency of platelet apheresis within the past 1 year, they were splitted into three groups: 6–11 times group (A), 12–17 times group (B) and 18–24 times group (C). Sample size for each group was calculated with directly count and each group had 32 regular plateletpheresis donors. The inclusion criteria for regular plateletpheresis donors were as follows: (1) the frequency of platelet apheresis within the past 1 year was ≥ 6 times; (2) the donor’s age was 18 to 55 years; (3) the donor’s body weight was more than 50 kg; (4) at before blood collection, the donor’s hemoglobin ≥ 120 g/L, hematocrit ≥ 0.36 and blood platelet count ≥ 150 × 10^9^/L; (5) the interval of platelet apheresis ≥ 2 weeks; and (6) All donors were not taken drugs for antiplatelet aggregation or inhibiting platelet metabolism (such as aspirin, Vit E, and penicillin) within 1 week and in a good healthy condition. Health examination was in accordance with the *Health examination criteria of blood donors* established by the Ministry of Health and Family Planning in China. The blood donors who participated in platelet apheresis for the first time during the same time period were as a control group (D group, totally 32 donors). Except that they had no history of platelet apheresis, the other criteria for inclusion were the same as the platelet apheresis group.

### Donor Baseline Information Collection

The characteristics of the donors was collected from the donors, including age, gender, experience of donation, frequency of donation and platelet collection amount.

### Specimen Collection

Two specimens were collected from every donor of all groups in each time donation. The first specimen was collected before plateletpheresis and the second specimen was collected after plateletpheresis completed within 15 to 20 min. Each specimen was separately stored in a 2 mL EDTA-K_2_ tube and sodium citrate tube, and taken for blood platelet count test and TEG test respectively. All tests were done by trained staffs.

### Blood Platelet Count

Blood platelet count (PLT), mean platelet volume (MPV) and platelet distribution width (PDW) were tested using a hematology analyzer (XS-800i, SYSMEX, Kobe, Japan) according to the manufacture’s instruction.

### Thromboelastography Test

A thromboelastography and auxiliary reagents (model 5000, Haemonetics Corp., Braintree, MA, USA) were used to detect coagulation function according to the manufacture’s instruction. In briefly, a volume of 1 mL anti-coagulated whole blood was collected and mixed with kaolin, and incubated for 4 min at room temperature. 340 µL of them was chosen and pipetted into a test cup containing 20 µL 0.2 M CaCl_2_, then the cup was mounted for the test. The test was completed within 15 min to 2 h, and the data were stored and analyzed using a processing software to obtain TEG images and reference values.

### Outcome Variables by TEG Test

Coagulation reaction time (R) refers to the time from the start of the test to the shed timing of 2 mm, which reflects the combined effect of all clotting factors and has a normal value of 3–8 min. Blood clot formation time (K) is the time from the completion of R detection to the shed timing of 20 mm, which reflects the results of the joint action of fibrinogen and platelets at the beginning of clot formation and has a normal value of 1–3 min. α-Angle is the angle between the horizontal line and the tangent line that is drawn from the clot formation point to the maximum curve curvature of the trace, i.e. the rate at which the blood clot reaches this intensity, which has a normal value ranging 53°–72°. The normal maximum amplitude (MA) is 50–70 mm, where cases with a MA < 45 mm are diagnosed as hypofunction or with a reduced number of platelets, and those with a MA > 72 mm are diagnosed with increased platelet function.

### Plateletpheresis Collection

Amicus cell separator and disposable consumable (C4R2316) were used for the platelet apheresis. The platelet number of each apheresis was set to 2.5 × 10^11^–5.0 × 10^11^. 2.2% sodium citrate was used for anticoagulation during the apheresis collection process at a ratio of 1:10 to whole blood.

### Statistical Analysis

Data is presented as number of cases and mean ± standard deviation (SD). Statistical analyses were performed using a SPSS 19.0 software (IBM Corp., NY, USA). The values of the parameters before and after plateletpheresis collection and different groups were compared with the two way ANOVA test. The correlations between the parameter of the TEG and frequency of platelet apheresis, PLT number were analyze using the Spearman’s correlation method. A difference with *p* < 0.05 was considered statistically significance.

## Results

### Donor Baseline Characteristics

The ratio of the male to female were 23:9, 24:8, 27:5 and 24:8 in the A, B, C, D groups respectively (Table 1). While the mean age were 40.7 ± 7.6, 39.8 ± 8.3, 40.2 ± 7.9 and 37.0 ± 9.3, platelet collection amount were 1.55 ± 0.37 U, 1.58 ± 0.38 U, 1.61 ± 0.33 U and 1.46 ± 0.31 U (2.5 × 10^11^ platelets numbers was defined as one unit) respectively in these A, B, C, D groups. The differences for ratio of the male to female, mean age, platelet collection amount were no significance among these four groups (*p* > 0.05). The frequency of plateletpheresis of the A, B, C, D groups were 8.2 ± 2.2, 14.4 ± 1.7, 20.6 ± 2.3 and 0, respectively (Table 1), the difference of them was significance (*p* < 0.05).

**Table 1:** Baseline data of the donor groups

Characteristics	6–11 times group (A group)	12–17 times group (B group)	18–24 times group (C group)	First time group (D group)	F test value	*p*
Male/Female	23/9	24/8	27/5	24/8	0.512	0.674
Age (years)	40.7 ± 7.6	39.8 ± 8.3	40.2 ± 7.9	37.0 ± 9.3	1.186	0.318
Platelet collection amount (U)	1.55 ± 0.37	1.58 ± 0.38	1.61 ± 0.33	1.46 ± 0.31	0.872	0.458
Platelet apheresis frequency (times/year)	8.2 ± 2.2*	14.4 ± 1.7*	20.6 ± 2.3*	0*	6.815	0.000

*Compared with the first time group, *p* < 0.05

### Comparison of the Values of the R Time, k Time, α-Angle, MA, PLT Count, MPV and PDW Before and After Plateletpheresis Donation in the A, B, C, D Groups

The values of the R time, k time, α-angle, MA, PLT count, MPV and PDW before and after plateletpheresis donation in the each group were listed in the Table 2. All of them were within the normal reference range.

**Table 2:** Comparison of platelet measurements before and after donation between donor groups

Variable	6–11 times group (A group)		12–17 times group (B group)		18–24 times group (C group)		First time group (D group)		Before/after comparison		Comparison between the groups	
	Before	After	Before	After	Before	After	Before	After	F	*p*	F	*p*
R (min)	7.02 ± 1.35	7.56 ± 1.37	7.09 ± 1.26	7.75 ± 1.37	6.88 ± 1.37	7.40 ± 1.30	6.89 ± 1.24	7.59 ± 1.34	13.49	0.000*	0.532	0.667
K (min)	2.23 ± 0.45	2.36 ± 0.51	2.23 ± 0.41	2.38 ± 0.44	2.24 ± 0.51	2.41 ± 0.57	2.16 ± 0.48	2.33 ± 0.50	6.56	0.011*	0.325	0.807
α-angle (°)	60.27 ± 5.1	58.73 ± 5.35	60.79 ± 4.49	58.40 ± 4.72	59.69 ± 4.90	58.02 ± 6.04	61.33 ± 5.25	59.22 ± 5.35	9.02	0.003*	0.814	0.487
MA (mm)	61.80 ± 3.47	58.85 ± 3.62	61.69 ± 3.47	58.54 ± 3.26	61.69 ± 4.08	58.42 ± 3.63	61.16 ± 4.12	58.75 ± 4.10	39.80	0.000*	0.128	0.944
PLT (× 10^9^/L)	234.4 ± 46.8	148.8 ± 30.3	246.3 ± 55.3	149.2 ± 31.2	241.4 ± 42.0	145.1 ± 25.8	238.0 ± 45.4	160.4 ± 34.7	317.27	0.000*	0.513	0.674
MPV (fl)	10.04 ± 1.06	10.25 ± 1.16	10.28 ± 1.01	10.25 ± 1.18	10.09 ± 0.68	10.00 ± 0.67	10.00 ± 0.94	10.02 ± 0.86	0.06	0.809	0.944	0.420
PDW (%)	11.65 ± 2.03	12.27 ± 3.27	12.08 ± 1.92	11.70 ± 2.28	11.64 ± 1.41	11.19 ± 1.27	11.61 ± 1.48	11.19 ± 1.56	0.41	0.524	1.510	0.212

*PLT* Blood platelet count, *MPV* mean platelet volume, *PDW* platelet distribution width, *R* coagulation reaction time, *K* clot formation time, *α* angle, *MA* maximum amplitude, *F* F test value, *p* probability value

*Significant differences between before and after plateletpheresis donation in each group were found, *p* < 0.05

Comparison the parameters of the before plateletpheresis donation and plateletpheresis completed in each group respectively, it was showed that the differences of the R time, k time, α-angle, MA, PLT count values between them were significant (*p* < 0.05). However, the differences of the MPV and PDW values between them were not significant (*p* > 0.05) in each group.

The difference values of the R time, k time, α-angle, MA, PLT count, MPV and PDW before plateletpheresis donation were statistically insignificant among the A, B, C, D groups (*p* > 0.05). The statistical parameters F (F-test value) and p values (probability value) were listed in the Table 2. The values of the R time, k time, α-angle, MA, PLT count, MPV and PDW after plateletpheresis donation were also compared among these four groups, all of them were not significant changed (*p* > 0.05, Table 2).

### Spearman Correlation Analysis

The coefficient of correlation and p values among frequency of plateletpheresis, PLT count, MPV, PDW with R time, k time, α-angle, MA were shown in Table 3. The number of PLT was significantly correlated with R time, k time, α-angle, MA parameters (*p* < 0.05). However, the frequency of plateletpheresis, MPV and PDW were not significantly correlated with R, k, α and MA parameters (*p* > 0.05), except for the correlation between the MA and PDW (*p* = 0.037).

**Table 3:** Correlation of coagulation parameters with frequency of donation and platelet parameters (n = 128)

Variable	R		K		α-Angle		MA	
	r	*p*	r	*p*	r	*p*	r	*p*
FD	− 0.049	0.444	0.015	0.809	− 0.055	0.384	0.035	0.576
PLT	0.255	0.000*	0.391	0.000*	0.359	0.000*	0.560	0.000*
MPV	− 0.100	0.113	0.035	0.581	− 0.012	0.855	0.119	0.059
PDW	− 0.123	0.050	0.030	0.632	− 0.028	0.659	0.132	0.037

*The number of PLT was significantly correlated with R time, k time, α-angle, MA parameters (*p* < 0.05), r: correlation coefficient value

## Discussion

Multiple plateletpheresis donations may be affected the donor health, including the coagulation function [5, 6, 10]. Routine blood coagulation testing in the plateletpheresis donations in the previously studies usually only included testing the activity of a single coagulation factor or assessment of prothrombin time (PT), activated partial thromboplastin time (APTT) and activated clotting time [5, 6, 10, 11]. Because conventional coagulation testing only reflected a certain segment of the coagulation process rather than making an overall assessment of coagulation function, the results were often controversial [5, 6, 10, 11]. Beyan et al. [10] reported that blood donors had a slight prolongation of PT time after apheresis, but there was no risk of hemorrhage. Akay et al. [11] found that antithrombin III was decreased after platelet apheresis and had a hypercoagulability risk. Now the application of TEG in monitoring coagulation function in apheresis donors has rarely been reported. TEG can comprehensively, completely and dynamically monitor the changes of coagulation function, and truly reflect the whole coagulation process. Therefore, it is rapidly popularized in the clinical coagulation function testing [12–15].

The previous studies mentioned that coagulation profile start to change around 30 min after procedures [5, 6, 10]. In our study, the plateletpheresis procedure was need nearly to 80 min to finish and the donor was rested at least for 15 min after plateletpheresis completed according to *Health examination criteria of blood donor* in China. Therefore, the second specimen was collected within the 15 to 20 min after plateletpheresis completed. In the present study, we used the TEG method for testing coagulation function in apheresis donors. It was found that R time was prolonged immediately after platelet collection. The main reason for prolongation of R time may be the binding of sodium citrate anticoagulant with calcium during the collection process, which prolonged the clotting time [16]. However, the R time is decreased by a small amplitude because the sodium citrate anticoagulant is rapidly metabolized and is unlikely to accumulate [17]. The average values of R time before and after the collection were still within the normal range in the regular and first time donors. Further studies found that the R time in the regular donor group did not show significant difference compared with the control group, and did not have significant correlation with the annual platelet apheresis frequency, which was similar to the result reported by Zhou [18]. Therefore, it can be concluded that even if there is a slight drop in coagulation function immediately after apheresis, this change is slight and transient, and is unlikely to cause severe changes in coagulation function that might lead to hemorrhage and is also unlikely to produce a long-term adverse effect on the coagulation function in the platelet apheresis donors.

K time is generally considered to be the result of the interaction of fibrinogen and platelets. α-angle mainly reflects the levels of coagulation factor and fibrinogen, and it is significantly related to K value. Cases with K > 4 min and/or α-angle < 45° are thought to have insufficient thrombin formation or fibrinogen hypofunction, and insufficient transformation of fibrinogen [19]. Yilmaz et al. [20] monitored the clotting factors in donors with dual platelet apheresis, and found that although factor V, VIII, IX, and fibrinogen were decreased after collection, they were still in the normal range, and PT and APTT did not significantly decrease. In the present study, the K time was prolonged, and the α-angle value was decreased after platelet apheresis, but they were still within the normal range. In addition, the K value and α-angle value in different frequencies of platelet apheresis groups were also within the normal ranges and did not show significant differences compared with the control group, which were suggested that a slight effect on the thrombin formation and fibrinogen function in these donors after platelet apheresis.

Most studies have shown that the number of platelets in a short period of time after apheresis is significantly reduced, but the platelets stored in the spleen through compensatory mechanisms of the body will be immediately released to the peripheral blood, while stimulating more bone marrow hematopoietic stem cells to be quickly differentiated and transformed into mature megakaryocytes, which are detached and enter the blood circulation. Hence, the number of platelets usually can be restored to the pre-collection levels about 4–6 days after platelet apheresis [21]. In the current study, the blood platelet count was significantly decreased immediately after the collection in the donor group. However, the blood platelet count of the regular donor groups before collection did not show significant difference compared with that of the control group. Although the results of the MPV and PDW of 6 to 11 times group were increased, however all of them were within the normal reference range and were not significance using the multiple comparison. Also, there was no significant correlation between the number of platelets and the annual frequency of platelet apheresis. Katz L et al. reported that regular platelet apheresis has no effect on the number of platelets in blood donors [6]. Platelet quality is not only related to quantity but also closely related to platelet function. It was reported that the platelet function was decreased to a certain extent after platelet apheresis using PFA-100 [22, 23], which made it difficult for the collected platelets to exert normal physiological functions. However, Zhou et al. [18] recently used TEG to detect platelet function and found that although the platelet function was slightly decreased 30 min after platelet apheresis, it recovered to pre-collection level after 7 days. MA is an important parameter reflecting platelet function in the TEG test. In the current study, the MA values of platelet apheresis donors were in the normal range and did not show significant differences compared with the control group, which indicated that regular platelet apheresis is unlikely to have a significant effect on the platelet function in the range of annual donation frequency permitted by national standards.

In summary, we have investigated the effect of frequent plateletpheresis on coagulation and platelet function of the donors in the Chinese population. Comprehensive monitoring of coagulation and platelet function in the platelet apheresis donors by means of TEG was showed that platelet apheresis was exerted a transient and mild effect on coagulation and platelet function in the blood donors. Comparison with the first time donors, regular donation did not have a significant and long-term adverse effect on the regular platelet apheresis donors, which was supported that the interval of plateletpheresis and the maximum frequency of annual blood donation were appropriate and valid in China. However, this study has some limitation. The sample size of the donor population was relatively small, and there was no followed up of the donors after the donation to investigate the degree of platelet recovery or the time taken to reach pre-donation levels. Larger studies in multiple centers would be provided more evidence to these results in the future. In additional, we did not analyze the effect of the donation interval on the coagulation function in donors and this need to study in the future.
